# Modified Medial Capsulorrhaphy Using Figure-of-Eight Suture in Hallux Valgus Correction Surgery

**DOI:** 10.1155/aort/8483004

**Published:** 2024-12-30

**Authors:** Raden Andri Primadhi, Ghuna Arioharjo Utoyo, Muhammad Naseh Sajadi Budi, Renaldi Prasetia

**Affiliations:** Department of Orthopaedics and Traumatology, Universitas Padjadjaran Medical School/Hasan Sadikin Hospital, Bandung, Indonesia

**Keywords:** capsulorrhaphy, figure-of-eight, hallux valgus, soft tissue procedure

## Abstract

**Objective:** Medial capsulorrhaphy is an important step in hallux valgus correction surgery; however, studies on this technique are limited. This study aimed to evaluate the viability and efficacy of modified medial capsulorrhaphy using figure-of-eight sutures for hallux valgus compared to the conventional technique.

**Methods:** Retrospective analysis was performed on patients with hallux valgus, comparing a group receiving standard longitudinal capsulorrhaphy (Group 1) and a group that underwent modified capsulorrhaphy using figure-of-eight sutures (Group 2). Basic anthropometry, preoperative hallux valgus angle (HVA), immediate postoperative HVA, and 6-month postoperative HVA were recorded. An independent sample *t*-test was performed to investigate differences between the groups.

**Results:** Thirty-four feet were enrolled in the study and divided into Group 1 (19 feet) and Group 2 (15 feet). There were no significant differences between the groups in terms of age, BMI, preoperative HVA, immediate postoperative HVA, and 6-month postoperative HVA (*p*=0.646, 0.752, 0.231, 0.792, and 0.933, respectively). However, we found that the average of HVA changes between immediate postoperative and 6-month postoperative was dissimilar (1.73 ± 2.37° Group 1 vs. 0.33 ± 1.29° Group 2, *p*=0.048)

**Conclusion:** While both techniques were efficacious in decreasing HVA, modified capsulorrhaphy using the figure-of-eight technique was better at maintaining corrected HVA until 6-month postoperative period. This technique is recommended because of its efficacy and simplicity.

## 1. Introduction

Hallux valgus (HV) is one of the most common complaints that bring patients to foot and ankle clinics, affecting an estimated 23% of adults aged 18–65 years and 35.7% of people aged over 65 years [1]. HV is still considered a mild yet disabling condition of the foot, presenting various symptoms such as a painful medial foot, abnormal walking patterns, balance impairment leading to fall risk, and severe foot deformity [1–3]. Other than genetic predisposition, anthropometry, and biomechanical alterations, lifestyle changes such as footwear choice also play a role in its pathomechanisms [4].

A common feature of HV is the lateral deviation of the great toe with a concomitant medial prominent first metatarsal head. The HV angle (HVA) and intermetatarsal angle (IMA) are conventional measurements that are widely accepted in clinical practice and surgical decision [5]. Management of HV should begin with nonoperative means, followed by surgical intervention when it fails. However, many patients visit the clinic in the advanced stages; therefore, surgery is the treatment of choice. In general, HV surgery involves osteotomy and soft-tissue procedures. HV involves not only an altered bone position but also the stretching or attenuation of soft tissue around the metatarsophalangeal joint (MTPJ). Both approaches should be carefully chosen to allow proper HV correction. Osteotomy typically corrects the IMA, whereas the distal soft tissue procedure corrects the HVA by restoring the physiological balance of the MTPJ capsule and ligamentous and muscular structures [6, 7]. Among various distal soft tissue procedures other than lateral soft tissue release, medial capsulorrhaphy plays an important role not only in deformity correction, but also in maintaining the corrected HVA [7]. Early postoperative relapse of valgus deformity is one of the determinants of treatment failure. Recurrence is related to inadequate soft tissue balance, including the strength of medial capsulorrhaphy.

Our literature review found that studies related to medial capsulorrhaphy are still limited when compared to other surgical procedures in HV correction surgery. One suture technique for capsular closure is the figure-of-eight technique which was reported effective in hip joint capsules [8]. Therefore, this preliminary study aimed to investigate the efficacy of modified medial capsulorrhaphy using a figure-of-eight suture technique in HV surgery, particularly in correcting and maintaining HVA.

## 2. Materials and Methods

Ethical clearance was obtained from the Hasan Sadikin Hospital Institutional Review Board (IRB) No. LB.02.01/X.6.5/183/2023 before commencing the study.

A retrospective analysis of patients who underwent surgical correction of HV between January 2018 and December 2022 was conducted. The inclusion criterion was mild to moderate deformity (HVA 20°–40°). The exclusion criteria were no considerable arthritic changes indicated for MTPJ fusion and prior scarring around the joint. All the patients underwent bunionectomy, lateral soft tissue release, corrective osteotomy, and medial capsulorrhaphy. To homogenize the surgical procedures, only patients who underwent lapidus fusion were enrolled in the study. A gauze folded lengthwise was wrapped around the great toe like a scarf for 4 weeks, drifting the hallux medially.

**Figure 1:**
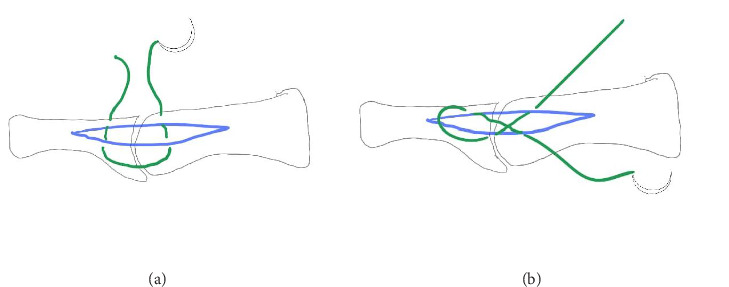
(a) Conventional longitudinal mattress suture; (b) figure-of-eight suture.

**Figure 2:**
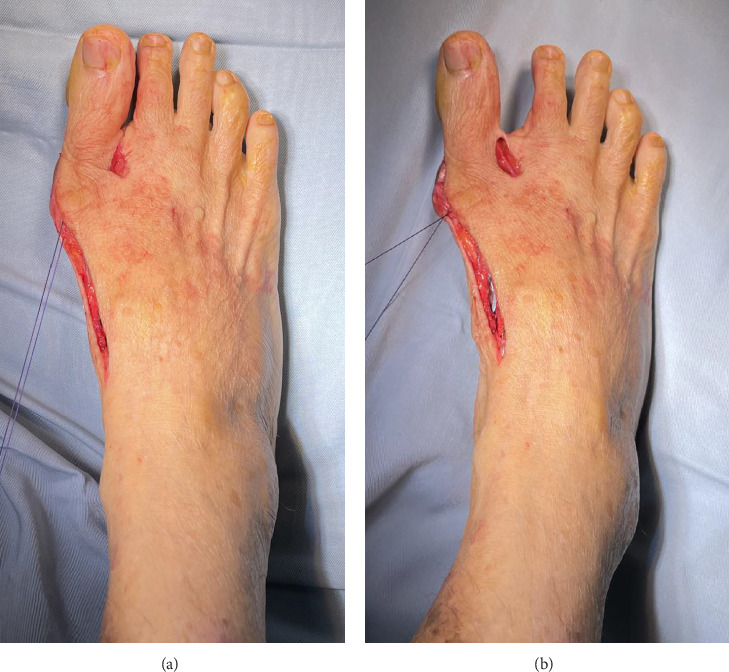
(a) Hallux lateral deviation before suture tightening; (b) corrected valgus deformity after suture tightening.

The patients were divided into two groups according to the selected medial capsulorrhaphy technique: standard longitudinal (Group 1) and modified capsulorrhaphy with figure of eight (Group 2), as illustrated in Figure 1. The suture was started at the proximal part of the MTPJ and then crossed to the opposite side to pierce the capsule at the distal part of the MTPJ. Subsequently, the suture continued transversely at the same level as the proximal phalanx of the hallux and then returned to the proximal part at the same level as the stitch starting point, thus completing a figure-of-eight configuration (Figure 2). Postoperatively, standard dressings were applied, with the great toe maintained in abduction position by using cohesive bandages encircling the great toe with or without toe spacers, for 3 weeks. The patients were allowed to partially bear weight using a rocker bottom footwear immediately postoperative as the pain tolerated.

**Figure 3:**
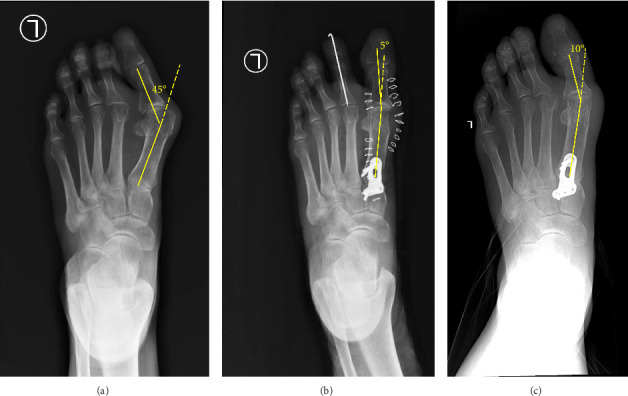
Dorsoplantar foot radiograph showing hallux valgus angle (HVA) measurements: (a) preoperative; (b) immediate postoperative; (c) 6-months postoperative.

Standard anthropometric measurements were carried out, including age, height, weight, and body mass index (BMI). HVA was obtained radiographically by measuring the angle between the lines that longitudinally bisect the proximal phalanx and the first metatarsal (Figure 3) [9]. Preoperative HVAs, immediately postoperative HVAs, 6-month postoperative HVAs, and HVA differences between immediate postoperative and 6-month postoperative were recorded. Differences between groups were examined and statistically analyzed with an independent sample *t*-test using IBM SPSS Statistics version 26.0 (Armonk, NY, IBM Corp).

## 3. Results

Thirty-four feet that fulfilled the inclusion criteria were included in this study. Nineteen feet were assigned to Groups 1 and 2, respectively. There were no significant differences in age (range 39–67 years, mean 55.03 ± 7.04), BMI (range 18.7–26.3 kg/m^2^, mean 22.07 ± 1.75), and preoperative HVA (range 23–55°, mean 36.67 ± 6.2) between groups (Table 1).

In view of the postoperative results, we found dissimilarities between the immediate postoperative results and 6-month postoperative follow-up results. The immediate postoperative HVAs were not significantly different between the groups, although a lower average HVA was observed in Group 1 (5.10 ± 5.29° Group 1 vs. 5.67 ± 7.04° Group 2], *p*=0.792) (Table 1). In the same way, HVAs measured in 6-month follow up were found not to be significantly different between groups (6.84 ± 5.05° vs. 6.67 ± 1.80°, *p*=0.933). We observed HVA discrepancies (Δ) between the immediate postoperative and 6-month postoperative, and found dissimilarity between the groups (1.73 ± 2.37° Group 1 vs. 0.33 ± 1.29° Group 2, *p*=0.048).

## 4. Discussion

The results revealed that the immediate postoperative results were comparable between the two groups but differed after 6 months. This means that both techniques are efficacious in obtaining and maintaining deformity correction along with other concomitant procedures. However, the modified techniques (Group 2) were superior when observed at 6-month follow-up visits, a period after soft tissue healing was achieved. While several patients had postoperative slight hallux varus, it was inconclusive whether this finding was related to the selected technique.

Postoperative HVA, along with preoperative HVA, IMA, and sesamoid position, are significant factors in HV recurrence [10, 11]. Hence, these problems must be addressed properly. Maintaining HV correction during this phase will likely determine the long-term surgical results. An association between immediate postoperative HVA achieved from surgery and HV recurrence [10].

The distal soft tissue procedure is an effective additional surgical procedure for HV correction that increases its corrective effect. The purpose of this procedure is to restore physiological and mechanical balance of the medial, lateral, dorsal, and plantar side structures around the 1^st^ MTPJ [12].

In a foot with HV deformity, the first metatarsal head shifts medially, resulting in medial ligamentous and capsular structure elongation, with consequent shortening of the lateral side structures. In addition to mediolateral imbalance, metatarsosesamoid luxation can occur and can be seen in a radiographic skyline view [12]. Biomechanical balancing by lateral release and medial tightening is crucial. Insufficient lateral release and medial capsule reefing lead to undercorrection or recurrence [12, 13].

In general, medial capsulorrhaphy is performed after completion of lateral structure release and metatarsal osteotomy. Thus, this step maintains alignment after correction. Longitudinal capsulotomy followed by elongated capsule resection and subsequent plication is a simple technique that does not risk capsule tears from excessive incisions. However, the mechanical direction of plication was not parallel to the intended correction. Many other medial capsulotomy techniques have been introduced, showing various results, such as L-shape, U-shape, Y-shape, and elliptic capsulotomy [14]. L-shape capsulorrhaphy are thought to be advantageous in plication by resecting the capsule excess to shift the flap to the proximal phalanges. However, Sever et al. compared standard longitudinal and inverted L-shaped capsulorrhaphy and reported that both techniques were effective in HV correction surgery without statistically significant differences between groups [15]. U-shaped capsulotomy was reported to be superior to inverted L-shaped capsulorrhaphy in maintaining the HVA and providing better ROM. This finding was attributable to the greater distance of the suture and the knots of U-shape capsulorrhaphy to the articular surface, compared with Y-shape capsulorrhaphy [6]. While the aforementioned techniques have been reported to be efficacious, problems may occur when the medial capsule is attenuated with loss of integrity due to the longstanding valgus deformity. In these cases, advancing the longitudinal incision in other directions may have caused the capsule to be torn. This research proved that simple longitudinal incision is sufficient when modified with the figure-of-eight capsulorrhaphy.

Several studies have reported the efficacy of using suture anchors to reinforce capsulorrhaphy as an adjuvant procedure; however, the extra cost might be a disadvantage [7, 16]. Internal brace is another option for enhancing medial capsulorrhaphy, particularly in severe HV presenting with a weakened medial capsule [17].

Figure-of-eight suture is reported effective in a capsular closure of tendon repair, enabling immediate movement with little risk of rupture or spacing of the suture [18]. Aoki et al. reported the use of figure-of-eight technique in hip capsular closure. The technique provided advantages including more robust capsular closure, watertight closure to minimize joint fluid leakage, and done following a systematical order that ensure appropriate closure [8]. On the other hand, the study reported that the torque to failure of simple stitch is comparable with figure-of-eight configuration [19]. However, the study used twice the number of the suture of simple stitch compared with figure-of-eight stitch. This suture was completed using the same suture material with standard longitudinal capsulorrhaphy. Other biomechanical advantage of this technique is that the hallux can be dynamically brought to varus by pulling the suture thread or the knot, which is not controllable using standard longitudinal capsulorrhaphy with mattress suture closure. The tightening force of this tensioning suture was parallel to the medial collateral ligament that can improve the joint's mechanical construct and produce the great toe abduction. In other ways, standard longitudinal capsulorrhaphy does not produce a dynamic correction, yet just maintains the correction achieved by spreading the toe or placing a spacer between great toe and second toe.

Period of 6 months postoperative was chosen for evaluation, as capsules generally heal in 6 months [20]. Wound maturation itself begins around the third week, followed by excess collagen degradation; thus, wound contraction reaches its peak [21].

The results reported herein should be considered in light of the following limitations: (1) it was a retrospective study with a small sample size; (2) the confounding effect of comorbidities such as generalized joint laxity was not analyzed; (3) sample bias might have resulted from the homogenized surgical procedure, specifically Lapidus fusion; (4) lack of previous studies on the topic, leading to an eventual literature gap; and (5) IMAs were not analyzed as variables. Despite the omission of IMAs from the variable list, this study was still relevant owing to the value of HVA measurement in determining the efficacy of medial capsulorrhaphy, considering that the IMA was identical between the two HVA follow-up evaluation periods.

## 5. Conclusion

This study showed that modified medial capsulorrhaphy with figure-of-eight sutures is effective in maintaining corrected HVA during the recovery period after HV surgical correction. This technique is simple, does not require additional incision, and does not require additional hardware. Nonetheless, these results must be interpreted with caution and several limitations must be considered. Further large-scale studies are required to validate our results.

## Figures and Tables

**Table 1:** Baseline and perioperative characteristics.

No	Variable	Group 1	Group 2	*p* value
1	Age (years)	54.5 ± 6.86	55.67 ± 7.45	0.646
2	BMI (kg/m^2^)	21.98 ± 1.31	22.18 ± 2.23	0.752
3	Preoperative HVA (°)	35.52 ± 6.34	38.13 ± 5.95	0.231
4	Immediate postoperative HVA (°)	5.10 ± 5.29	5.67 ± 7.04	0.792
5	4 weeks postoperative HVA (°)	6.84 ± 5.05	6.67 ± 1.80	0.933
6	HVA difference (4) and (5) (°)	1.73 ± 2.37	0.33 ± 1.29	0.048

## Data Availability

The data that support the findings of this study are available from the corresponding author upon reasonable request.
